# Exploiting cancer’s phenotypic guise against itself: targeting ectopically expressed peptide G-protein coupled receptors for lung cancer therapy

**DOI:** 10.18632/oncotarget.18403

**Published:** 2017-06-07

**Authors:** Mahjabin Khan, Tao Huang, Cheng-Yuan Lin, Jiang Wu, Bao-Min Fan, Zhao-Xiang Bian

**Affiliations:** ^1^ Laboratory of Brain-Gut Research, School of Chinese Medicine, Hong Kong Baptist University, HKSAR, Kowloon Tong, P.R. China; ^2^ YMU-HKBU Joint Laboratory of Traditional Natural Medicine, Yunnan Minzu University, Kunming, P.R. China; ^3^ State Key Laboratory of Applied Organic Chemistry, College of Chemistry and Chemical Engineering, Lanzhou University, Lanzhou, P. R. China

**Keywords:** cancer, G-protein coupled receptors, lung cancer, overexpressed receptors, peptides

## Abstract

Lung cancer, claiming millions of lives annually, has the highest mortality rate worldwide. This advocates the development of novel cancer therapies that are highly toxic for cancer cells but negligibly toxic for healthy cells. One of the effective treatments is targeting overexpressed surface receptors of cancer cells with receptor-specific drugs. The receptors-in-focus in the current review are the G-protein coupled receptors (GPCRs), which are often overexpressed in various types of tumors. The peptide subfamily of GPCRs is the pivot of the current article owing to the high affinity and specificity to and of their cognate peptide ligands, and the proven efficacy of peptide-based therapeutics. The article summarizes various ectopically expressed peptide GPCRs in lung cancer, namely, Cholecystokinin-B/Gastrin receptor, the Bombesin receptor family, Bradykinin B1 and B2 receptors, Arginine vasopressin receptors 1a, 1b and 2, and the Somatostatin receptor type 2. The autocrine growth and pro-proliferative pathways they mediate, and the distinct tumor-inhibitory effects of somatostatin receptors are then discussed. The next section covers how these pathways may be influenced or ‘corrected’ through therapeutics (involving agonists and antagonists) targeting the overexpressed peptide GPCRs. The review proceeds on to Nano-scaled delivery platforms, which enclose chemotherapeutic agents and are decorated with peptide ligands on their external surface, as an effective means of targeting cancer cells. We conclude that targeting these overexpressed peptide GPCRs is potentially evolving as a highly promising form of lung cancer therapy.

## INTRODUCTION

Lung cancer is a daunting malady victimizing millions globally, on an annual basis. Being the most common form of tumor worldwide, 1.82 million lung cancer cases were diagnosed and 1.59 million deaths occurred, in 2012 [1]. The widespread nature and high global incidence of lung cancer calls for urgent advances in treatment regimes.

The two main subtypes of lung cancer are non-small cell lung cancer (NSCLC) and small cell lung cancer (SCLC). NSCLC is a heterogeneous group of tumors comprising adenocarcinoma, squamous cell carcinoma, and large cell carcinoma. Whilst occurrence of histological type differs among countries, NSCLC is more prevalent than SCLC [1]. Of lung cancer diagnoses globally, adenocarcinoma has the highest incidence (29–69%), followed by squamous cell carcinoma (28–46%), and then SCLC (9–22%), with the lowest frequency [2].

Existing first-line and conventional therapies for lung cancer include chemotherapy, radiation therapy, surgery, and most recently, immunotherapy. With the toxicities and health hazards associated with existing treatments, novel therapies are increasingly gaining attention. In recent years, meticulous studies coupled with technological innovations have enabled the scientific community to better understand intricate intracellular and extracellular events at the genomic, molecular and cellular levels within neoplasms [3]. This has led to the evolution of a novel form of cancer treatment, called targeted therapy, that has maximum toxicity for cancer cells and minimal toxicity for healthy cells. It is directed towards exploiting the abnormal composition of cell surface receptors on cancer cells. Agents disrupting angiogenesis through vascular endothelial growth factor receptor (VEGFR) signaling and those targeting the epidermal growth factor receptor (EGFR) have enhanced outcome in NSCLC in large and randomized trials, thus, paving the entry to clinical practice [3]. The agents involved, typically monoclonal antibodies or small molecules, are directed towards the ectopically expressed proteins/receptors in various intracellular processes essential for disease/tumor perpetuation and growth [3]. The therapeutic potential of one of the most important ectopically expressed receptors in cancer, the G-protein coupled receptors (GPCRs), has yet to be explored.

GPCRs, the largest family of cell surface receptors, play crucial roles in both normal and diseased body states [4]. They have been implicated to play vital roles in the formation and progression of tumor, and have been shown to be ectopically expressed on the cell surface of cancer cells [5, 6]. GPCRs have served, for over a millennium, as targets for drug intervention in the treatment of disease; medicinal drugs used by the Romans and Egyptians contained alkaloids and opioids, derived from foxglove and mandrake, which moderate their action through GPCRs [7]. Due to their significance in the many physiological processes, GPCRs are now a major focus of current pharmaceutical research: about 50% of drugs on market target the G-protein coupled receptor family [6].

With over 800 members [8], GPCRs are categorized into five major classifications, namely Rhodopsin (class A); Secretin and Adhesion (class B); Glutamate (class C); Adhesion (Class D) and Frizzled/smoothened (Class E) (TAS2) receptor families. These families are further divided into many subfamilies based on sequence similarity and ligand classes, including peptide, opsin, prostaglandin, MECA, Melatonin receptors, etc. The peptide receptor subfamily is the pivot of the current article owing to its significant association with the neoplastic state and its strict selectivity and high specificity to its cognate ligands. Research has proven the high specificity and potency of peptide-based therapeutics, which has intensified the efficacy of rather nonspecific drugs and reduced drug-associated toxic side-effects. Furthermore, peptide-based therapeutics have facilitated diagnosis and noninvasive gross morphological evaluation of solid tumors. Radiotherapy utilizing peptide-based compounds has been proven effective in the treatment of certain forms of solid tumors. [9]. For these reasons, peptide-based therapeutics such as peptide-drug conjugates [10–12] and peptide ligand functionalized nanoparticles [13, 14], among others, are popular compounds undergoing scrutiny and evaluation in ongoing oncological research.

This review highlights some recent GPCR-targeted therapeutics tested as potential candidates for lung cancer treatment. The article begins with the introduction of various overexpressed peptide GPCRs in lung cancer and their significance in neoplastic processes in tumor development. The next two sections describe how these processes may be altered or ‘corrected’ by targeting the overexpressed GPCRs through treatment system involving agonists and antagonists, as well as through Nano-scaled delivery platforms that enclose chemotherapeutic drug(s) and are decorated with peptide ligand of the peptide GPCR to be targeted, on their external surface. Finally, future directions and perspectives will be discussed.

### Overexpressed peptide receptors in lung cancer

Common overexpressed peptide GPCRs in lung cancer include the Cholecystokinin B/Gastrin receptor, the Bombesin receptor family, Bradykinin B1 and B2 receptors, Arginine Vasopressin Receptors 1a, 1b and 2, and Somatostatin receptor type 2. All these receptors belong to the rhodopsin-like class A family of the GPCR superfamily [15–18]. Almost all of them are overexpressed in both, NSCLC and SCLC, with Table 1 showing the extent of over- or ectopic expression in different subtypes of lung cancer. All the cognate peptide ligands are classed as neuropeptides. Cholecystokinin (CCK), gastrin, Gastrin releasing peptide (GRP), Neuromedin-B (NMB), bradykinin and vasopressin are considered growth-stimulatory neuropeptides as they act as autocrine/paracrine growth factors [19, 20] to promote cellular proliferation [21] in lung cancer and other cancers, whereas somatostatin is classified as a growth-inhibitory neuropeptide.

**Table 1:** Ectopically expressed peptide GPCRs in lung cancer

Receptor	Gene	Lung cancer subtype	Patient tissue
Cholecystokinin-B/ Gastrin receptor	CCKBR/ CCK2	SCLC	mRNA overexpressed in 10/10 (100%) [24] and protein overexpressed (57%) [34]
		Squamous cell carcinoma	mRNA overexpressed in 1/13 (7.7%) [24]
		Adenocarcinoma	mRNA overexpressed in 1/21 (4.76%) [24]
Bombesin receptor subtype 3	BRS3/ BB3/ BB3R	SCLC	mRNA overexpressed in 4/9 (44.4%) [50]
		NSCLC	N/A
		Bronchial carcinoid	mRNA overexpressed in 9/26 (34.6%) [50]
		LCNEC	mRNA overexpressed in 1/1 (100%) [50]
Gastrin Releasing Peptide Receptor (GRPR)	GRPR/ BB2/ BB2R	SCLC	mRNA overexpressed in 3/9 (33.3%) [50]
		NSCLC	N/A
Neuromedin-B Receptor	NMBR/ BB1/ BB1R	SCLC	N/A
		NSCLC	N/A
Bradykinin Receptor B1	BDKRB1/ B1BKR/ BKR1/ bradyb1	Adenocarcinoma	Protein overexpressed in 6/6 (100%) [70]
		Squamous cell carcinoma	Protein overexpressed in 5/6 (83.3%) [70]
		Large cell carcinoma	Protein overexpressed in 5/6 (83.3%) [70]
		Small cell carcinoma	Protein overexpressed in 6/6 (100%) [70]
		Carcinoid tumors	Protein overexpressed in 4/6 (66.6%) [70]
Bradykinin Receptor B2	BDKRB2/ BK-2	Adenocarcinoma	Protein overexpressed in 6/6 (100%) [70]
		Squamous cell carcinoma	Protein overexpressed in 6/6 (100%) [70]
		Large cell carcinoma	Protein overexpressed in 4/6 (66.6%) [70]
		Small cell carcinoma	Protein overexpressed in 3/6 (50%) [70]
		Carcinoid tumors	Protein overexpressed in 5/6 (83.3%) [70]
Arginine Vasopressin Receptor 1a	AVPR1A/ V1aR	SCLC	mRNA overexpressed in 5/7 (71.4%) [96]
		NSCLC	mRNA overexpressed in 17/22 (77.3%) [96]
Arginine Vasopressin Receptor 1b	AVPR1b/ V3R/V1bR	SCLC	mRNA overexpressed in 2/7 (29%) [96]
		NSCLC	mRNA overexpressed in 4/22 (18%) [96]
Arginine Vasopressin Receptor 2	AVPR2/ V2R	SCLC	mRNA overexpressed in 7/7 (100%) [96]
		NSCLC	mRNA overexpressed in 18/22 (82%) [96]
Somatostatin receptor (type 2A)	SSTR2	SCLC	Protein overexpressed in 23/61 (37.7%) [128]
		Typical carcinoid	Protein overexpressed in 17/24 (70.8%) [128]
		Atypical carcinoid	Protein overexpressed in 37/73 (50.7%) [128]
		LCNEC	Protein overexpressed in 20/60 (33.3%) [128]

### Cholecystokinin-B/Gastrin receptor (CCKBR)

The cholecystokinin-B/gastrin receptor (CCKBR) is activated by its endogenous ligands, cholecystokinin and gastrin. Under physiological conditions, the CCKBR is commonly expressed in the stomach, pancreas and in particular areas of the human brain [22–24]. The receptor exerts a growth-stimulating effect in peripheral tissues [25, 26] and is involved in gastric acid secretion [27]. In the central nervous system, the receptor mediates emotional behaviors such as pain, anxiety and panic [28–31].

The CCKBR is, however, not expressed in the normal lung [24]. The mRNA expression was below the detectable level in all 12 normal lung tissues [24]. However, CCKBR mRNA was detectable in lung cancer, including SCLC, adenocarcinoma and squamous cell carcinoma [24]. CCKBR has been proposed as an attractive therapeutic target specifically for SCLC, whose prognosis persists to be disappointing despite initial response to chemotherapy [24]. Receptor autoradiographic studies revealed that CCKBR is expressed in high percentages in lung [24] and pancreatic [32] cancers, medullary thyroid carcinomas, some ovarian cancers, astrocytomas, gastrointestinal tumor, and colorectal cancer [33–35]. CCKBR was either not expressed or rarely expressed in differentiated thyroid cancers, meningiomas, lymphomas, renal cell cancers, and prostate carcinomas [34]. In regards to lung cancer, CCKBR is expressed in both SCLC and NSCLC [24, 34] (Table 1). Recently, Tripathi *et al.* created a comprehensive, literature-based map elucidating intracellular signaling cascades mediated by CCKBR (and CCKAR) [36]. The map may assist in the formulation of novel hypotheses on molecular mechanisms [36] and aid in the discovery and identification of novel molecular markers for CCKBR-based cancer therapeutics.

The paralog of CCKBR, CCKAR, is rarely or negligibly expressed in SCLC [37, 38] and other tumors [34, 39], with its expression level being significantly less than that of CCKBR. Thus, CCKAR is not covered in the current review.

### Bombesin receptor family (GRPR, NMBR, and BRS-3)

Three receptors have been identified to belong to the bombesin (BN) receptor family, namely Gastrin Releasing Peptide receptor (GRPR), the Neuromedin-B receptor (NMBR), and the Bombesin receptor subtype 3 (BRS-3). High-affinity endogenous ligands for GRPR and NMBR are gastrin releasing peptide (GRP) and neuromedin-B (NMB), respectively; meanwhile, BRS-3 remains an orphan receptor, having low affinity for all natural-occurring bombesin type peptides [40, 41]. Human GRP is the mammalian analog of bombesin (BN), a 14 amino-acid peptide primarily discovered in the skin of the frog Bombina bombina [42].

The BN receptor family is a family of brain-gut peptide receptors [42, 43]. BRS-3 has high homology, 47–51% to GRPR/NMBR. Under physiological conditions, the BN-like peptides act on the central nervous system to regulate food intake, body temperature and glucose levels and certain behavioral responses [44]. In the periphery, GRP and NMB are involved in a spectrum of actions including smooth muscle contraction and endocrine/exocrine secretion. GRP is named for its property to induce gastrin release from gastrin (G) cells in the antral mucosa. GRPR has been fully characterized in the gastrointestinal epithelial cells [45, 46] and plays a role in gastrointestinal functions [47]. BRS-3 and its undefined ligand mediate vital metabolic and endocrine processes [48]. The activation of the BN receptor family causes numerous intracellular signaling cascades, which are chiefly moderated by phospholipase-C activation causing stimulation of protein-kinase C and cellular calcium changes [40, 42, 49]. Nevertheless, much is yet to be discovered on the BN receptor family.

Overexpression of the BN receptors has been evidenced in several tumor types, including lung, breast, prostate, intestinal, pancreatic, and colon carcinomas, gastrinomas, neuroblastomas, pituitary adenomas, head and neck cancers, and tumors of the CNS (gliomas, meningiomas) [42, 50–55]. Lung cancer has been the principal experimental model for discovering possible treatments curing through BN receptor family targeting. This is because SCLC has long been known to produce and release BN-related peptides [53, 54]: in 1985, SCLC was the first human tumor in which an autocrine growth effect was discovered [56], revealing fundamental information on the indispensability of this group of peptides and their cognate receptors. Overexpression of the BN receptor family was shown in different subtypes of lung cancer [50] (Table 1).

Generally, human tumors preferentially express the individual receptor subtypes of the BN receptor family, especially more frequently GRPR and less frequently NMBR [57, 58]. The significance of the bombesin/GRP-R in promoting cancer cell growth through the induction of autocrine loops and the high density of these receptors on the cell surface of various human tumors renders the receptor family a popular focus of nuclear oncology and extremely attractive targets for developing innovative therapeutic strategies, particularly for life-threatening neuroendocrine tumors such as SCLC [59–62].

### Bradykinin receptors B1 and B2 (B1R and B2R)

Two pharmacologically distinct kinin receptor subtypes exist, namely bradykinin receptors B1 (B1R) and B2 (B2R) which are mainly involved in pain and inflammatory pathways [63]. The endogenous ligands for B2R are bradykinin (BK) and lysyl-bradykinin (Lys-BK) [17]; those for B1R are metabolites lacking the C-terminal arginyl residue, [des-Arg9] BK and [Lys-des-Arg9] BK [17, 64]. B2R is ubiquitously and constitutively expressed, whereas the physiological expression of B1R is extremely low. However, the expression of B1R surges in stressful situations [65], such as various pathological conditions, in several cell types including neurons, endothelial and blood cells, and smooth muscles. B1R is induced in various models of cancer, angiogenesis, inflammation, pain syndromes, diabetes mellitus, multiple sclerosis, epilepsy, and Alzheimer’s disease [17, 66–69].

B2R and B1R are highly expressed in cancers of the lung [70], breast [71], prostate [72], gall bladder [73], head and neck squamous cell carcinoma (HNSCC) [74], chondrosarcomas [75], colorectal adenomas [76], clear cell renal carcinoma [77], esophageal squamous cell carcinomas, and astrocytic tumors [78]. Table 1 shows the upregulation of B1R and B2R in lung cancer subtypes [70].

Whereas B1R and B2R may have a high expression profile in numerous tumor types, their relative expression levels may differ in different tumors. For instance, Chee *et al.* reported a somewhat similar expression pattern of the two bradykinin receptor subtypes in lung cancer [70], whereas a study by Nicoletti *et al.* on glioma cells showed a higher expression of B1R relative to B2R [79]. The authors of the glioma study attribute the higher expression level of B1R to the inducible nature of the receptor [79].

Studies on mice lacking each receptor gene and different specific peptidic and non-peptidic antagonists have implied that both B1R and B2R are potential therapeutic targets in various pathophysiological events in the aforementioned diseases [17]. Due to negligible expression of B1R in healthy tissues, the receptor is very appealing as an imaging marker for tumor diagnosis and for the development of anticancer therapeutic agents [80, 81].

### Arginine Vasopressin Receptors 1a, 1b and 2 (V_1a_, V_1b_, and V_2_)

Three arginine vasopressin receptors (AVPRs) have been identified via molecular cloning techniques, V_1a_ (vascular), V_1b_ (pituitary) and V_2_ (renal) [82]. The endogenous ligand for the AVPRs is the nonapeptide amine, vasopressin (or antidiuretic hormone), produced by magnocellular neurons of the hypothalamus [83]. This hormone is essential for survival as it is involved in the fundamental physiological processes of osmotic and cardiovascular homeostasis [84].

The expression of each subtype of AVPR is distinct and tissue-specific. V_1a_ receptors are normally expressed in the heart, brain, testis, liver, superior cervical ganglion, vascular smooth muscle cells and renal medulla [85, 86]. V_1a_ receptors in the brain moderate anxiety producing responses to arginine vasopressin, whilst their presence in the vasculature help moderate the pressor actions of arginine vasopressin through a phospholipase C-mediated pathway [87, 88]. The V_1b_ receptor is involved in mediating anxiety and stress in humans and rats [89]. Their presence in the anterior pituitary helps mediate the ACTH-releasing effects of AVP, through a phospholipase C-mediated pathway [90]. V_1b_ receptors are also present in extra-pituitary tissues such as the adrenal medulla, kidney and brain [91]. On the other hand, the V_2_R is mostly expressed in the renal medulla, where it is involved in mediating the antidiuretic effect of AVP [85, 92].

In normal cells, the vasopressin gene is transcriptionally restricted, while in SCLC, it is activated concomitantly with expression of all three of its receptors (V_1a_R, V_1b_R, and V_2_R) [93–95]. Vasopressin receptors are overexpressed only in lung cancer [96, 97] and ACTH-secreting pituitary tumors [98, 99]. Table 1 shows the pattern of overexpression of AVPRs in lung cancer subtypes [96, 97].

### Somatostatin receptor type 2 (SSTR2)

The past 30 years saw the identification and characterization of the somatostatin receptor family, which includes five different subtypes, SSTR1- SSTR5 [100–102], that show 40–60% structure homology [103]. The endogenous high-affinity ligand for the SSTRs is the acid polypeptide, somatostatin, which is native to the central nervous system and various peripheral tissues and organs. Somatostatin has several biological functions including the potent inhibition of basal and stimulated secretions from a wide range of exocrine and endocrine cells [102, 104], and acts as a neurotransmitter (exerting both stimulatory and inhibitory effects [105]) and neuromodulator in the central nervous system, and as an antiproliferative agent for cell proliferation and differentiation [106], and as an autocrine/paracrine agent in the immune system [107]. SSTRs are differentially expressed in the immune and central nervous systems, pituitary, thyroid and adrenal glands, gut, pancreas and kidney [108, 109]. Multiple receptor subtypes may be co-expressed in a tissue-specific pattern, having distinct physiological roles [108]. In the peripheral nervous system, SSTRs are implicated to play roles in nociception [110]. In the pituitary gland, SSTR2 is involved in the release of ACTH, GH, and TSH [102].

SSTRs are highly expressed in a variety of tumors, including cancers of the lung [111, 112], breast [113, 114], prostate [115], brain [116, 117], and pheochromocytomas [118], gastric carcinomas [119, 120], meningiomas [121], hepatocellular carcinoma [122], endocrine pancreatic tumors, gastrointestinal carcinoids, and thyroid medullary cancer [123]. Out of the five subtypes, the SSTR type 2 is generally the most commonly overexpressed receptor in cancer [108, 124–127]. Thus, SSTR2 is the subtype covered in the present article. The extent of SSTR2 overexpression in lung cancer subtypes [128] may be viewed in Table 1.

### Carcinogenesis pathways mediated by overexpressed peptide GPCRS

Cancer cells produce and secrete the neuropeptides CCK/gastrin [34, 129], GRP and NMB [130, 131], bradykinin [132], and vasopressin [133, 134]. A plethora of evidence exists elucidating the autocrine growth and pro-proliferative effects exerted by CCK/gastrin [135, 136], GRP and NMB [137, 138], bradykinin [139, 140] and vasopressin [21, 94, 95, 141, 142] through their respective receptors. In the process, they increase the colony number of cancer cells, including lung cancer [21, 135, 143]. Weber *et al.* reported that even activation of orphan receptor BRS-3 may contribute to cancer cell proliferation, since the analogue [d-Phe^6^, β-Ala^11^, Phe^13^, Nle^14^] bombesin (6–14) caused enhanced nuclear oncogene expression, MAPK phosphorylation, and Elk-1 activation in lung cancer cells [144]. Research has indicated that GPCR transactivation of epidermal growth factor receptor (EGFR) is significantly involved in cancer cell proliferation [135, 145]. For instance, the BRS-3 agonist (DTyr^6^, βAla^11^, Phe^13^, Nle^14^)bombesin^6–14^ (BA1) caused Tyr^1068^ phosphorylation of EGFR in NCI-H727 or NCI-H1299 cells stably transfected with BRS-3 (NCI-H1299-BRS-3), and significantly increased the clonal growth of NCI-H1299-BRS-3 cells. Other BRS-3 agonists such as (DTyr^6^, R-Apa^11^, Phe^13^, Nle^14^)bombesin^6–14^ (BA2) and (DTyr^6^, R-Apa^11^, 4-Cl,Phe^13^, Nle^14^)bombesin^6–14^ (BA3) also caused EGFR transactivation in NCI-H1299-BRS-3 cells [146]. Furthermore, it has been found that CCK/ gastrin [135, 147–150], GRP [53, 54, 131, 138, 151, 152], bradykinin [153, 154] and vasopressin [21, 155, 156] bind with high affinity to their cognate receptors to promote DNA synthesis [157], increase intracellular calcium levels [37, 132, 143, 147–150, 153–155, 158], promote cellular growth, proliferation, survival [139, 140, 159–165], cause loss of cell adhesion, and stimulate tumor progression, invasion, migration and metastasis [166–169]. Moreover, it is thought that CCK/gastrin [170], GRP and NMB [130, 131, 171], and bradykinin [172, 173], by acting on their cognate receptors, promote angiogenesis and suppress apoptosis.

Somatostatin stands out from the crowd of receptors included in this review. While all ligands whose cognate receptors included in this paper act as growth factors, somatostatin is inherently a broad inhibitory neuropeptide and has anti-secretory, anti-proliferative and anti-angiogenic activities [174]. It mediates its effects through five receptors but SSTR2 subtype is generally the most commonly expressed in neoplastic cells [123]. Somatostatin and its analogs/agonists inhibit tumor growth and metastatic spread through activation of SSTRs on both cancer and microenvironment cells, such as endothelial cells of tumor vessels that are responsible for the neovascularization of the tumor. SST exerts its effects through direct antiproliferative (inhibition of mitogenic stimuli of growth factors such as IGF-1 and cell cycle arrest) and proapoptotic signals, as well as, indirectly, by inhibiting the secretion of proliferative and angiogenic growth factors and hormones, or suppressing neoangiogenesis at the endothelial cell level or regulating an immune response [175, 176].

### Therapeutics targeting overexpressed peptide receptors for lung cancer

Standard therapies have encountered a plateau in improving patient overall health and survival and quality of life [177]. In addition, despite initial responsiveness to existing standard cancer treatments, like chemotherapy and radiotherapy, some cancer types like SCLC are highly aggressive and commonly relapse within months [178]. Cancer therapeutics targeting overexpressed and/or ectopically expressed peptide GPCRs in cancer is one novel and promising treatment option which has gained considerable interest over the past two decades. The ectopic and over-expression of GPCRs such as CCKBR, GRPR, B1R, and other receptors, drives tumor growth. Therefore, interference in receptor signaling and inhibition of signaling pathways driving tumor growth and perpetuation are envisioned as ideal targets for cancer therapeutics. This may be achieved using monoclonal antibodies, agonists and antagonists. This section will include some of the recent targeted therapeutics, for each peptide GPCR, that have been discovered to show positive results *in vitro* and *in vivo* to ultimately contribute to tumor shrinkage.

### Cholecystokinin-B/Gastrin receptor (CCKBR)

A plethora of studies have shown the significant role that upregulation of CCKBR and its endogenous ligands play in the regulation of tumor growth and maintenance [25, 26]. Antagonists for CCKBR help downregulate expression of the receptor resulting in decreased DNA synthesis, cell cycle arrest through inhibition of G(1) to S phase progression, reduced cancer cell proliferation, mobility and invasiveness. In addition, downregulation of CCKBR increased caspase-3 activity and TUNEL-positive cells, suggesting apoptotic activity [179].

**Figure 1:**
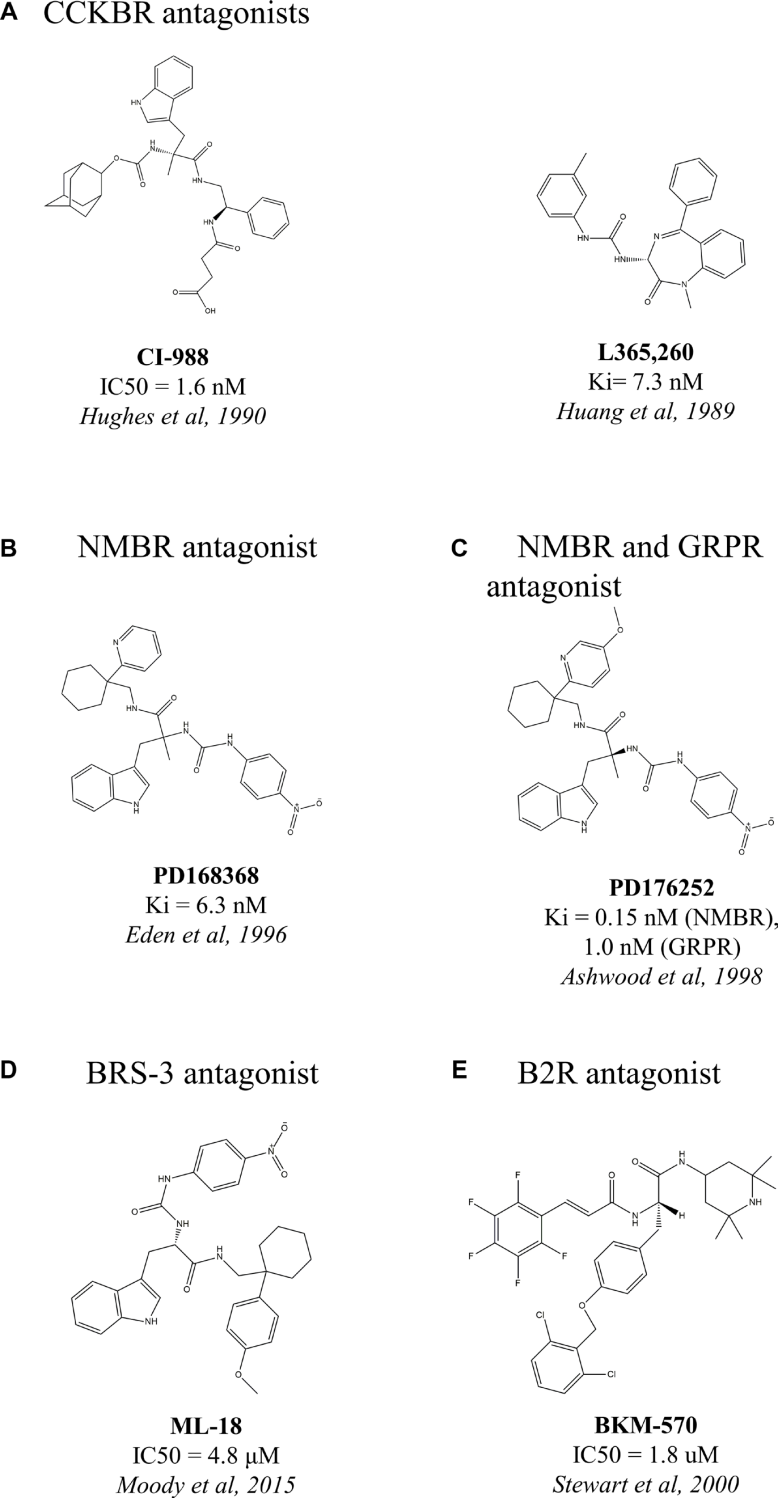
Chemical structures of non-peptide antagonists of overexpressed peptide GPCRs in lung cancer

CCKBR therapeutics include antagonists such as CI-988 (Figure 1A, [180]) and L365, 260 (Figure 1A, [181]). CI-988 inhibited the abilities of CCK-8 to elevate cytosolic Ca^2+^, to stimulate EGFR, ERK and FAK tyrosine phosphorylation as well as VEGF expression, and so cell growth and proliferation in NCI-H727 lung cancer cells [135]. L365, 260 inhibited the proliferative capacity of human medullary thyroid carcinoma cells, resulting in a marked attenuation in growth [182].

### Bombesin Receptors (GRPR, NMBR, and BRS-3)

Bombesin related peptides are synthesized and secreted by cancer cells and are reported to cause autocrine-growth effects in human SCLC [56] and other cancers [50, 183, 184]. Some studies demonstrated that monoclonal BN antibodies inhibit the growth of these tumors both *in vitro* and *in vivo* xenografts [56]. Many studies demonstrate the potential therapeutic significance of BN receptor antagonists and other agents that inhibit the growth-stimulatory effect of BN receptor agonists on tumors [61].

High-affinity NMB receptor non-peptide antagonist PD168368 (Figure 1B, [185]) inhibited lung neoplastic cell growth by inhibiting the transactivation of EGFR and the tyrosine phosphorylation of ERK caused by NMB-like peptides [152].

High affinity NMBR (K_i_ = 0.15 nM) and moderate affinity GRPR (K_i_ = 1.0 nM) non-peptide antagonist PD176252 [186] (Figure 1C, [186]) significantly inhibited NCI-H1299 proliferation (and was more potent than PD168368) and considerably inhibited lung cancer colony number *in vitro* [187]. PD176252 inhibited the binding of GRP/bombesin to GRPR and blocked the ability of the ligand to elevate cytosolic calcium levels and c-fos mRNA in lung cancer cells NCI-H1299, NCI-H345 and H1299. It also blocked the ability of bombesin to cause tyrosine phosphorylation of focal adhesion kinase in NCI-H1299 cells [187]. *In vivo* studies revealed that the antagonist dose-dependently inhibited NCI-H1299 xenograft growth in nude mice [187].

Both the afore-mentioned antagonists, PD168368 and PD176252, do not bind BRS-3 with high affinity [188]. For BRS-3, however, the novel BRS-3 antagonist ML-18 (Figure 1D, [189]), had anti proliferative effects in lung cancer [189]. ML-18 inhibited specific (125)I-BA1 (DTyr-Gln-Trp-Ala-Val-βAla-His-Phe-Nle-NH2)BB(6–14) binding, with an IC_50_ value of 4.8 μM, to lung cancer cells NCI-H1299 stably transfected with BRS-3. ML-18 bound to GRPR and NMBR with lower affinity with IC_50_ values of 16 and > 100 μM, respectively. ML-18 inhibited the ability of BA1 to increase cytosolic calcium and tyrosine phosphorylation of EGFR and ERK in lung cancer cells [189].

Another BRS-3 antagonist, BRS-3 ant. (DNal-Cys-Tyr-DTrp-Lys-Val-Cys-Nal)NH_2,_ blocked BA1-induced EGFR or ERK tyrosine phosphorylation in lung cancer cells and diminished clonal growth of NCI-H1299-BRS-3 cells [146]. In addition, BA1, BA2, BA3 (BRS-3 agonists) and BRS-3 ant. (BRS-3 antagonist) blocked specific ^125^I-BA1 binding to NCI-H1299-BRS-3 cells with IC_50_ values of 1.1, 21, 15 and 750 nM, respectively [146].

An additional antagonist of BN/GRP is the powerful inhibitor RC-3940-II. It significantly inhibited growth of H460 and A549 NSCLC xenografts and caused the upregulation of tumor suppressor gene p53, which may contribute to the anti-tumor effects of the antagonist [190], and an increase in several angiogenesis inhibitors and a reduction in proangiogenic genes [191]. There was also an escalation in the number of cells with lower G(0)/G(1) DNA content and in those blocked in S and G2/M phases [192]. Zhou *et al..* reported on antibody-mediated therapy for SCLC. They conjugated a monoclonal antibody OKT3 (anti-CD3) with a bombesin/GRP antagonist (Antag2) to create a bispecific molecule, OKT3xAntag2. The molecule was shown to mediate growth inhibition and apoptosis of SCLC cells by activated T cells via the activation and cleavage of Poly (ADP-ribose) polymerase (PARP) and caspase-3 *in vitro* and *in vivo* [193].

### Bradykinin receptors B1 and B2 (B1R and B2R)

In 1984, the Stewart laboratory discovered the first antagonists for bradykinin (BK) and ongoing investigations by this group and others resulted in the present generation of extremely potent peptide antagonists that are orally active and have proven resistant to all tested degrading enzymes [194, 195]. It is noteworthy that prolonged administration of bradykinin antagonists to humans has not caused adverse effects [196].

CU201 (also called B9870) is a highly potent, metabolism-resistant bradykinin antagonist peptide dimer. CU201 is a growth inhibitor for SCLC both *in vitro* [197, 198] and, *in vivo* for SCLC SHP-77 and NSCLC A549 tumor growth [199], giving significant inhibition (65%) of tumor growth in athymic nude mice models upon daily intratumoral administration at a dose of 5 mg/kg/day [195]. The antagonist inhibited proliferation signals but induced apoptotic signals [197] by a novel “biased agonist” action. It blocked the G_αq_, G_11_ signaling pathway that induces intracellular free Ca^2+^ in response to bradykinin but stimulated the G_α12_, G_13_ pathway associated to c-JUN kinase activation [44], inducing caspase-3 activity and causing unique changes in apparent nuclear DNA binding, ultimately resulting in cell death. Therefore, CU201 offers great promise in being branded as a new form of targeted therapy for neoplasms with neuroendocrine properties, owing to its unique mechanism of action [197].

BKM-570 (Figure 1E, [200]) is the non-peptide mimetic of CU201 [198]. BKM-570 strongly inhibited extracellular signal-regulated kinases 1/2 (ERK1/2) and protein kinase B (AKT) [201], effectively suppressing tumor cell growth [202]. BKM-570 showed strong anticancer activity against SCLC and prostate cancer *in vivo* [198] and *in vitro* [195] and various other cancer cell lines of NSCLC, cervix and colon cancers *in vitro*, where the antagonist did not harm the growth of normal pulmonary fibroblasts [195].

Another bradykinin antagonist is R954. It is a stable, selective and potent peptide antagonist of the inducible B1R. It exhibits favorable preclinical pharmacological, pharmacokinetic characteristics and toxicological profile [64] and has shown antitumoral activity on ascitic and solid tumors induced by Ehrlich cell inoculation in rats and mice [203]. However, more research is warranted for this antagonist as current evidence is greatly limited. R954 has, nevertheless, been tested in some other conditions with success, including acute lung injury [204], osteoarthritis [205], inflammatory edema [206] and more. The testing of this antagonist in cancer models is relatively recent when compared to its testing in other pathological states, hence the limited data on its effects in cancer. This antagonist may potentially emerge as a new anti-cancer drug.

### Arginine vasopressin receptors 1a, 1b and 2 (V_1a_, V_1b_, and V_2_)

Vasopressin agonists with anticancer effects exert their action through V_2_ receptor, as it is involved in antiproliferative effects; the V_1_ receptors are related to cellular proliferative effects [207]. The synthetic nonapeptide 1desamino8Darginine vasopressin, desmopressin (dDAVP), is an agonist on the V_2_ receptor. dDAVP decreased expression levels of neuroendocrine markers chromogranin A (CgA) and specific neuronal enolase (NSE) in aggressive SCLC cell line NCI-H82 [208]. dDAVP also displayed dose-dependent anti-metastatic effects with maximum impact at clinically relevant doses of 1–2 μg/kg [209] and a 70% reduction in the number of pulmonary nodules in experimental lung metastatic disease [210]. dDAVP seemed to stimulate a dual angiostatic and antimetastatic effect, breaking the cooperative function between cancerous and endothelial cells during residual tumor progression [210, 211]. Pastrian *et al.* showed that the amino acids present at the loop of dDAVP are crucial for the antiproliferative activity of dDAVP, accentuating the significant role of the N terminal region of the peptide in the interaction with the cancer cell surface receptor. These findings help show novel strategies for designing and developing improved compounds with augmented stability for tumor therapy [212]. An analog of dDAVP, [V^4^Q^5^]dDAVP , decreased tumor growth and angiogenesis in F3II mammary tumour-bearing immunocompetent mice, and exhibited higher antimetastatic efficacy than dDAVP on experimental lung colonisation by sarcomatoid mammary carcinoma F3II cells [213]. Preliminary acute toxicology studies revealed that [V^4^Q^5^]dDAVP was well-tolerated at doses ≥ 300-fold above those needed for anti-angiogenic/antimetastatic effects [213]. DDAVP and [V^4^Q^5^]dDAVP markedly decreased proliferation, doubling time, and migration in NCI-H82 cells. [V^4^Q^5^]dDAVP demonstrated a greater cytostatic effect than dDAVP, on cellular proliferation in the NCI-H82 cell line [208].

### Somatostatin receptor type 2 (SSTR2)

Somatostatin is of limited clinical utility owing to its extremely short half-life of 2–3 minutes, resulting in the development of many synthetic somatostatin analogs (SSAs) such as octreotide, lanreotide and pasireotide, by attenuation of the polypeptide chain while preserving binding affinity to the SST receptors [214]. Octreotide and lanreotide bind to SSTR-2 with high affinity (0.32 and 0.5 nM) [215], whilst pasireotide binds SSTR2 with 2.5 times lower binding affinity than octreotide [216]. Subsequently, long-acting release (LAR) octreotide [217] and long-acting release (LAR) lanreotide [218] were also developed.

Octreotide and lanreotide are registered in many countries [219] and have proven to be successful therapeutics in the management of tumors. These SSAs have similar effects, in neuroendocrine tumors, to native endogenous somatostatin in that they decrease cellular proliferation, induce apoptosis, inhibit protein synthesis and cell signaling [220], and inhibit secretory processes [221].

SSAs exert antiproliferative effects through direct and indirect mechanisms. Direct mechanisms entail the activation of SSTRs in tumor cell surfaces [222]. Upon activation, SSTRs induce cell cycle inhibitors such as p21, p27 and p130/Rb, thus resulting in cell cycle arrest [222]. Somatostatin and SSAs can directly induce apoptosis in cancer cells, with a p53- dependent or independent mechanism [223]. SSTR2, along with SSTR1, SSTR3 and SSTR4, may be involved in inhibiting cell invasion by impacting the PI3K pathway. SSTR2 is also important in restoring gap junctions, which are essential for contact inhibition and maintenance of a differentiated condition [224, 225]. Indirect mechanisms by which SSAs exert antiproliferative effects involve the inhibition of circulating growth factors such as insulin-growth factor (IGF), vascular endothelial growth factor (VEGF), growth hormone (GH), platelet-derived growth factor (PDGF), basic fibroblast growth factor (bFGF), as well as inhibition of tumor angiogenesis by inhibiting the proliferation and migration of endothelial cells and monocytes, which secrete proangiogenic factors [226–229]. It is thought that the main mechanism of angiogenesis inhibition may revolve around interference in endothelial NO release. [230].

Whilst the antiproliferative and anti-tumor effects of SSA were shown *in vitro* and *in vivo* years ago, their use as anti-cancer agents has only recently been recognized [224]. Evidence of SSA success in lung neuroendocrines is very limited; a mere handful studies have analyzed the antiproliferative effects of SSAs in bronchial carcinoids. A retrospective study involving 48 patients with lung NET discovered that the most frequently administered first-line therapy in patients with advanced disease, was administration of SSAs [231]. A phase III RADIANT-2 trial randomized 429 patients with hormonally active carcinoid tumors, including bronchial NETs, to treatment with octreotide LAR plus placebo or octreotide LAR plus anticancer agent, everolimus. Analysis of the subgroups revealed that, for the 44 patients with lung carcinoid who received only the SSA as active drug, the median PFS was 5.6 months [232]. Furthermore, tumor shrinkage, although not satisfying RECIST criteria for partial response, was seen in 27% of participants from the octreotide LAR monotherapy cohort [233]. The results of a randomized phase III trial (PROMID) demonstrated that the median time to progression in patients with midgut carcinoid tumors treated with octreotide LAR was 14.3 months versus 6 months in patients treated with placebo [234]. Recently, a retrospective study analyzed the efficacy of octreotide in 15 patients with advanced pulmonary carcinoids, and reported a median PFS of 15 months and a 70% disease control rate [235]. A prospective, randomized, open-label, 3-arm, phase II study evaluating the effectiveness and safety of pasireotide, everolimus, or both, in patients with advanced neuroendocrine carcinoma of the lung and thymus (LUNA trial) is currently being conducted in Europe [236].

### Targeting overexpressed peptide receptors through delivery systems for lung cancer

Targeted drug delivery (TDD) platforms are based on the success and advantages of using peptides to successfully target over- or ectopically expressed receptors in cancer cells. Specific tumor receptor interaction is instrumental in mediating the ability of peptide ligand-cytotoxic constructs to induce cytotoxicity [237]. TDD platforms may involve peptide-drug conjugates (PDCs) that generally comprise a therapeutic moiety, a linker moiety, and a peptide as a targeting moiety [238]. Also, external surfaces of Nano-sized drug delivery systems, including liposomes and micelles, are decorated with peptide receptor ligands. The use of peptides as targeting moiety is advantageous because they cause the cytotoxic drug-loaded delivery platform to be targeted specifically to tumor cells, inflicting no/negligible damage to healthy cells. This section focuses on some recent works incorporating receptor peptide ligands within Nano-scale delivery systems for targeted and effective therapeutic intervention in neoplasms. A summary of the works covered in this section may be viewed in Table 2.

**Table 2:** Ligands of ectopically expressed peptide receptors in targeted delivery/imaging systems for lung cancer

Receptor	Ligand	Ligand sequence	Used in targeted delivery/ imaging system
Cholecystokinin-B/Gastrin receptor	CCK-33	KAPSGRMSIVKNLQNLDPSHRISDRDYMGWMDF-NH_2_ [http://www.uniprot.org]	N/A
	CCK-8	DYMGWMDF-NH_2_ [241]	N/A
	CCK-5	GWMDF-NH_2_ [http://www.uniprot.org]	N/A
	Gastrin	pEGPWLEEEEEAY(SO_3_H)GWMDF-NH_2_ [268]	N/A
Bombesin Receptor Subtype 3 (BRS-3)	Orphan receptor (has synthetic ligand)	[D-Tyr, β-Ala, Phe,Nle] BB(6–14) [269]	N/A
Gastrin Releasing Peptide (GRPR)	Gastrin releasing peptide (GRP)	VPLPAGGGTVLTKMYPRGNHWAVGHLM-NH_2_ [268]	N/A
	Bombesin peptide	pEQRLGNQWAVGHLM-NH_2_ [268]	PTXPEGBBN[7–13] [247]
Neuromedin-B receptor (NMBR)	Neuromedin B (NMB)	GNLWATGHFM-NH_2_ [268]	N/A
Bombesin Receptor family (BRS-3, GRPR, NMBR)	Pan-BBN ligand (binds to all 3 receptors)	D-Tyr, β-Ala, Phe, Nle] BBN [6–14] [246]	CPT-L2-BA3 [237, 246]
Bradykinin Receptor B1	Bradykinin	RPPGFSPFR- NH_2_ [268]	N/A
Bradykinin Receptor B2			
	Bradykinin Potentiating Peptide (BPP)	EWPRPQIPP- NH_2_ [251]	Pt-CS-BPP [251]
Arginine Vasopressin Receptor 1a	Vasopressin	CYFQNCPRG-NH_2_ [268]	99mTc(NS3)(CN-AVP) [257]
Arginine Vasopressin Receptor 2			99mTc(NS3)(CN-AVP(an)) [257]
Somatostatin receptor (type 2)	Somatostatin	AGCKNFFWKTFTSC-NH_2_ [268]	N/A
	Octreotide (analog of somatostatin)	(D)FCF(D)WKTCT-ol [270]	1.[OCT(Phe)-PEG-ss-PTX] [259]
			2. SSTR2-3207-86 [261]

### Cholecystokinin-B/Gastrin receptor (CCKBR)

The Cholecystokinin-B/gastrin receptor binds CCK-33, CCK-8, CCK-5 and gastrin. However, it is CCK-8 that is most commonly used as a targeting moiety in targeted delivery systems. There is a lack of research and classical literature focusing on CCK-8 labelled/loaded delivery systems tested specifically in lung cancer. They were however, tested in other cell types including HuVEC cells and A431 epidermoid carcinoma cell line, with positive results [239–241].

### Bombesin receptors (GRPR, NMBR, and BRS-3)

Due to the high frequency of overexpression of BN receptor subtypes in many tumors, increasing interest has led to massive investments of resources and time towards investigating the Bombesin receptors as potential therapeutic targets. *In vitro* autoradiographic studies reveal that GRPR, out of the three human BN receptors, is the most commonly expressed in human tumors [242]. Hence the analogue of GRP, the Bombesin peptide (QQRLGNQWAVGHLM) is a popular targeting ligand used in targeted therapy directed at GRP receptors. The eight-residue C-terminal peptide sequence (BN[7–14]), may be used to selectively target GRPR. Several studies show that the BN (7–14) fragment, modified with radiometal complexes on its N-terminus, preserves its affinity for these receptors, hence proving vital for diagnostic or therapeutic nuclear medicine applications [243–245].

Moody *et al.* discovered a potent BN agonist drug conjugate (CPT-L2-BA3) that had cytotoxic effect towards cells overexpressing all mammalian BN receptor subtypes [237, 246]. MTT and clonal growth assays revealed that CPT-L2-BA3 inhibited the growth of NSCLC NCI-H1299 cells and H1299 xenograft growth in nude mice, showing a high cytotoxic effect [237, 246]. On cells lacking BN receptors, CPT-L2-BA3 had only a very small effect. CPT-L2-BA3 also inhibited growth of several other tumor cell lines, including lung cancer (NCI-H69), neuroblastoma (IMR32, SKNSH), glioblastoma (U-87MG), leukemia (MOLT-4), breast cancer (MCF-7), prostate cancer (PC-3, DU-145, LNCaP), gastric cancer (Hs746T) and colon cancer (HT-29) cell lines with IC_50_ values ranging from 33 to 2269 nM [237]. Safavy *et al.* showed that the cytotoxicity of PTXPEGBBN[7–13], a tumor directed derivative of paclitaxel, was improved by a factor of 17.3 for 24 h and 10 for 96 h exposure times, in comparison to paclitaxel alone in NCI-H1299 human NSCLC cells [247]. The IC_50_ of the conjugate was lower than that of the free drug by a factor of 2.5 for exposure times of both 24 h and 96 h [247]. Moreover, the order of addition of peptide ligands to nano-sized delivery systems is an appealing and noteworthy perspective since the order may affect the size, stability and cytotoxicity exhibited by the final product. Post-bombesin decorated nanostructured lipid carriers (NLC) exhibited more stability and markedly higher transfection efficiency and better anti-tumor activity than pre-bombesin decorated NLC for lung cancer therapy, both *in vitro* and *in vivo* [248].

### Bradykinin B1 and B2 receptors (B1R and B2R)

Bradykinin has a short plasma half-life of about 15 seconds [249] and is rapidly inactivated in pulmonary circulation [250]. Therefore, bradykinin potentiating peptide (BPP) has been used as targeting moiety for targeted delivery [251]. BPPs have the ability to inhibit bradykinin inactivation in lung [252] and potentiate bradykinin action [253–255]. BPP has a relative selectivity for tumor vasculature and exerts potent effects through B2R, which is commonly overexpressed in various types of tumors [70, 256]. Wang *et al.* speculated that BPP can promote drug accumulation in primary tumor and lung metastasis by facilitating an increase in vascular permeability and enhancing drug penetration [251]. They used a 9 amino acid residues-long BPP (EWPRPQIPP) and a drug which was bioreductive sensitive platinum (IV) compound which became cisplatin in the intracellular reductive environments. Both the drug and the BPP were covalently attached to 120 nm-diameter chitosan nanoparticles. *In vivo* biodistribution and tumor inhibition investigations revealed that, compared with the free drug and the peptide-free nanoparticle formulation, the BPP-studded nanoparticle formulation boasted superior efficacy in promoting drug accumulation in tumor, thus confining tumor growth and prolonging the lives of tumor-bearing mice. In addition, drug accumulation in lung metastasis was about 17% and 20% injected dose/gram of lung for the chitosan nanoparticles without and with BPP, respectively. This was 10-fold greater than that of free cisplatin, which was about 1.6% injected dose/gram of lung. Thus with improved drug accumulation in lung metastasis tissue, the BPP-studded chitosan nanoparticle formulations effectively inhibited metastasis to lungs [251].

### Arginine vasopressin receptors 1a, 1b and 2 (V_1a_, V_1b_, and V_2_)

To our knowledge, the arginine vasopressin peptide has not been used as a targeting ligand in delivery systems carrying cytotoxic chemotherapeutic agents. The nonapeptide has, however, been conjugated to radionuclides for tumor imaging/diagnosis. Gniazdowska *et al.* investigated the conjugation of vasopressin (AVP) (CYFQNCPRG) and its analogue (d(CH2)5[D-Tyr(Et2), Ile4, Eda9 ]AVP (AVP(an)) to technetium-99m radionuclide to test its potential as a diagnostic radiopharmaceutical for SCLC patients [257]. In serum, the 99mTc(NS3)(CN-AVP) was enzymatically degraded into two species, whilst the 99mTc(NS3)(CN-AVP(an)) conjugate proved to be highly stable. In addition, the AVP (an) is one of the many effective antagonists to V2 receptor [258] and conjugates containing the AVP (an) demonstrated specific and high binding ability to V2 receptors on the SCLC cell line H69 [257].

### Somatostatin receptor type 2 (SSTR2)

Recently, Yin *et al.* developed a redox-sensitive prodrug, octreotide(Phe)-polyethylene glycol-disulfide bond-paclitaxel [OCT(Phe)-PEG-ss-PTX] for the targeted intracellular delivery of paclitaxel [259]. The conjugate demonstrated approximately 23,000-fold increase in water solubility than the parent paclitaxel [259], which has extremely low water solubility [260]. The OCT(Phe)-PEG-ss-PTX was selectively internalized into tumor cells through SSTR-mediated endocytosis and showed a high degree of cytotoxicity and apoptosis-inducing ability against NCI-H466 SCLC cells that ectopically expressed SSTR. Furthermore, *in vivo* studies on NCI-H466 tumor-bearing nude mice showed that the OCT(Phe)-PEG-ss-PTX had superior tumor-targeting ability and antitumor activity along with minimal collateral damage, compared with free paclitaxel [259]. Also, recently, Redko *et al.* developed five novel PDCs by separately linking the SSTR2 specific backbone cyclic peptide 3207–86 with five different anticancer drugs. The PDCs exhibited selective and significant cytotoxic effects in the human NSCLC cell line H1299 and various other human cancerous cell lines overexpressing SSTR2 [261].

Shen and coworkers analyzed the anti-tumor effects of a conjugate developed by coupling two molecules of paclitaxel to octreotide in A549 human NSCLC cells xenografted into nude mice [262]. 2paclitaxel-octreotide caused significant tumor growth inhibition at 150 nM/kg and 300 nM/kg, and significantly lengthened the tumor doubling time and considerably decreased tumor micro vessel density at these doses. Increased amount of fragmented DNA was seen in the 2paclitaxel-octreotide single and double dose groups relative to the controls [262]. Similarly, Sun *et al.* developed paclitaxel-octreotide conjugates which dose- and time-dependently inhibited the growth of NSCLC cells A549 and Calu-6. Paclitaxel and the conjugates could stimulate the increase of G(2)/M phase ratio in A549 cells [263]. A noteworthy point is that the conjugates had less cytotoxicity than paclitaxel-alone in SSTR-negative fibroblasts.

## CONCLUSIONS AND PERSPECTIVES

Overexpressed peptide GPCRs are valuable biomarkers for cancer diagnosis, imaging, and treatment. While CCKBR [24], and B1R [264] are not expressed in normal lungs but only in cancerous lungs, all the other receptors included in the present review are expressed in both normal lungs as well as cancerous lungs; however they are upregulated in cancerous lungs. In this regard, CCKBR and B1R are ideal therapeutic targets for lung cancer because minimal side-effects can be expected. In contrast, the expression of other receptors in normal tissues must be considered when targeting ligands are assessed for use as imaging or therapeutic agents. In addition, normal tissue samples from the primary tumor organ site should be scrutinized prior to a receptor’s designation as an overexpressed or upregulated entity, and normal tissues from multiple organ sites for concern for toxicity should be examined prior to the determination of a marker as an appropriate entity for the ligand targeting of therapeutic agents, or for concern for background signal interference prior to the targeting of imaging agents [265].

It is likely that, except somatostatin, all neuropeptides mentioned in this article bind to their overexpressed cognate receptors on cancer cell surfaces to establish an autocrine loop by inducing growth of the tumor cells they originate from, promoting neoplastic growth. Using peptide and nonpeptide antagonists to interrupt this autocrine loop has been proven an effective approach for the inhibition of tumor growth *in vivo* as well as *in vitro* in preclinical studies. In the coming years, these anti-autocrine therapies may be tested alone in clinical trials to determine the optimal dose for humans and evaluate the safety profile, or with immunotherapies to generate synergistic effects. For peptide antagonists, due to the inherent serum instability and renal infiltration, modifications such as cyclization or PEGlyation could be applied to improve their pharmacokinetics profile.

With high affinity and specificity, peptide ligands of overexpressed GPCRS are good carriers to deliver cancer-toxic agents. Since most studies involving the targeted delivery of cytotoxic agents were performed in various tumor types excluding lung cancer, future studies could investigate peptide-conjugated nanocarriers/targeted delivery systems in lung neoplasm [266]. For example, besides using vasopressin as targeting peptide in radiopharmaceuticals for tumor imaging and diagnosis, the use of the peptide in targeted delivery system for therapeutic purposes is deemed scientifically viable and appropriate for tumors overexpressing vasopressin receptors [267]. Therefore, the incorporation of vasopressin and/or its agonists to targeted delivery systems could be a future step for research and development of targeted therapy for cancer.

With the highest mortality rate worldwide and the various concerns associated with existing treatments, lung cancer requires urgent attention from the scientific community. Targeting peptide GPCRs that are ectopically expressed in lung tumor, qualify as promising candidates for lung cancer treatment in the near future. The successful results obtained from the use of SSAs in growth hormone-secreting tumors bears witness to this insight.

## Abbreviations

SCLC: Small cell lung cancer
NSCLC: Non-small cell lung cancer
GPCR: G-protein coupled receptor
CCK: Cholecystokinin
CCKBR: Cholecystokinin-B/Gastrin receptor
GRP: Gastrin releasing peptide
GRPR: Gastrin Releasing Peptide receptor
NMB: Neuromedin-B
NMBR: Neuromedin-B receptor
BRS-3: Bombesin receptor type 3
BPP: Bradykinin potentiating peptide
B1R: Bradykinin receptor 1
B2R: Bradykinin receptor 2
AVPRs: Arginine vasopressin receptors
V1aR: Vasopressin receptor 1a
V1bR: Vasopressin receptor 1b
V2R: Vasopressin receptor 2
SSTR2: Somatostatin receptor type 2
SSAs: Somatostatin analogs
VEGFR: Vascular endothelial growth factor receptor
EGFR: Epidermal growth factor receptor
IGF: Insulin-growth factor
GH: Growth hormone
PDGF: Platelet-derived growth factor
bFGF: basic fibroblast growth factor
LAR: (Octreotide/Lanreotide) long acting release
TDD: Targeted drug delivery
PDC: Peptide drug conjugate
